# Effect of Carbonation Treatment on the Properties of Steel Slag Aggregate

**DOI:** 10.3390/ma16175768

**Published:** 2023-08-23

**Authors:** Jian Ma, Guangjian Dai, Feifei Jiang, Ning Wang, Yufeng Zhao, Xiaodong Wang

**Affiliations:** 1 College of Architecture and Civil Engineering, Jiangsu University of Science and Technology, Zhenjiang 212000, China; mj198004@mail.ustc.edu.cn (J.M.); daigj1230@163.com (G.D.); 18205255126@163.com (X.W.); 2 Suzhou Institute of Technology, Jiangsu University of Science and Technology, Suzhou 215600, China; 18862615220@163.com (N.W.); 17625216718@163.com (Y.Z.); 3 College of Civil Engineering, Nantong Institute of Technology (NIT), Nantong 226000, China

**Keywords:** steel slag, carbonation, expansion, volume stability, waste management, sustainability

## Abstract

Steel slag is the waste slag generated after steel smelting, which has cementitious activity. However, untreated steel slag can damage the integrity of steel slag concrete due to its harmful expansion. This study prepared porous aggregates by mixing powdered steel slag, fly ash, and cement and carbonated them with CO_2_ under high pressure conditions (0.2 MPa). The effect of carbonation on the performance of steel slag aggregate was studied using volume stability and crushing value. The effect of different carbonation conditions on the products was studied using X-ray diffraction (XRD) and thermogravimetric (TG) analyses, and the carbon sequestration efficiency of steel slag under different treatment methods was quantitatively evaluated. The research results indicate that untreated steel slag was almost completely destroyed and lost its strength after autoclave curing. With the increase in temperature and carbonation time, the performance of steel slag aggregate gradually improved and the pulverization rate, expansion rate, and crushing value gradually decreased. According to the experimental results of XRD and TG, it was found that the reaction between f-CaO (free CaO) and CO_2_ in steel slag generated CaCO_3_, filling the pores inside the aggregate, which was the internal reason for the improvement of aggregate performance. After comparison, the best carbonation method was maintained at 55 °C for 72 h. After carbonation, the steel slag aggregate had a pulverization rate of 2.4%, an expansion rate of 0.23%, a crushing value of 23%, and a carbon sequestration efficiency of 11.27% per unit weight of aggregate.

## 1. Introduction

With the rapid development of global industrialization, the harm of global climate change is increasing day by day. With the development of ecological civilization construction in China, carbon emissions and carbon neutrality have received great attention. The Chinese government solemnly promised in 2020 to achieve the “Dual Carbon” Goal of carbon peaking by 2030 and carbon neutrality by 2060 [1,2]. Currently, relevant organizations and institutions around the world, including the World Meteorological Organization (WMO), the European Integrated Carbon Observing System (ICOS), and the China Meteorological Administration, are monitoring greenhouse gases, predicting climate change trends, and proposing carbon reduction measures. Reducing carbon emissions and protecting the environment has gradually become a human consensus [3,4,5].

As a major country in the steel industry, the output of China’s iron and steel has steadily occupied the first place in the world for many years. With the rapid development of the steel industry, a large amount of steel slag has been generated during the production of coarse steel [6]. At the same time, the steel industry produces a significant amount of CO_2_ in its production. It is estimated that its CO_2_ emissions in 2019 were about 2.6 billion tons, accounting for 7% of global energy system emissions [7]. Because of the complex composition of steel slag, the utilization ratio of steel slag is always low. At present, the utilization ratio of steel slag in China is only about 30% [8] due to the complexity of steel slag compositions with a high concentration of f-CaO and f-MgO [9]. Steel slag is widely used in road construction [10]. Cansu Kurtulus et al. [11] successfully synthesized foam glass from ferrochrome slag and waste soda-lime glass for the first time. Because of the high output and low consumption of steel slag, a large amount of steel slag can not only occupy a lot of land resources but also cause pollution to the surrounding soil and air [12,13]. Some scholars have studied the reuse of steel slag, and now steel slag is mainly used as aggregate for roadbed landfill [14,15], agricultural fertilizer [16,17], and wastewater treatment [18,19], but they have not been used on a large scale.

Due to the fact that concrete is the most commonly used building material, some researchers have attempted to use steel slag to prepare steel slag concrete. Carlo Pellegrino [20] used steel slag to replace natural aggregate to prepare the concrete, as well as the mechanical properties and durability of steel slag concrete. Zhuang et al. [21] used steel-slag-modified ultrafine blast furnace slag as an admixture to prepare high-strength concrete and studied its ultra-high temperature and self-shrinkage issues; the results showed that steel slag can effectively retard the main hydration heat release peak, decrease the accumulated heat, and reduce the growth rate of self-shrinkage with regard to cement–ultrafine blast furnace slag composite binder. M. Fernández Bertos et al. [22] found that carbonation can improve the physical and chemical properties of steel slag aggregate and can also fix CO_2_ in the steel slag, which provides a new way for the utilization of steel slag. Wang et al. [23] studied the compaction and carbonation of gypsum, steel slag, and water; based on microscopic analysis, the results demonstrated that gypsum can promote the compression strength of steel slag and the absorption of carbon dioxide, and the main hydration products are C-S-H phase and ettringite, while the main carbonation products are calcite and monocarbonate. Liu et al. [24] studied the effects of curing conditions on the mechanical properties of steel slag–cement cementitious materials at high temperatures and under carbonation. The results show that the case after carbonation can effectively prevent the corrosion of cement-based materials, improve the durability of cementitious materials, and enhance the compactness of cementitious materials. Shi et al. [25,26,27] studied the results of an experiment using carbonation curing to improve the performance of recycled aggregates to quantify the degree of CO_2_ curing of recycled aggregates by assessing the percentage of carbonation of the aggregates. It was found that with the increase in carbonation curing time, the strength of the aggregate and the degree of CO_2_ curing increased. After carbonation curing, the aggregate shows a more compact microstructure. The formation of calcium carbonate and silica gel greatly reduces the porosity; thus, the aggregate has higher strength. Mo Liwu et al. [28] used a 99.9% concentration of CO_2_ to cure aggregates at a pressure of 0.1MPa and studied the carbonation technology for treating steel slag to prepare concrete, improving its strength and stability [29,30,31]. The abovementioned research has explored the potential application of steel slag in concrete, recognized the impact of steel slag stability, and demonstrated that carbonation curing of steel slag can improve the volume stability of steel slag. However, further research on how to improve carbonation efficiency and accurately calculate carbon sequestration is needed. And due to the density of steel slag being about 3.2 g/cm^3^, which is much greater than the 2.5 g/cm^3^ density of stones, the weight of steel slag concrete is greater than that of ordinary concrete, which increases the load on the structural foundation. The light weight of steel slag concrete is also an important factor hindering its engineering application.

This study uses a mixture of fly ash and steel slag powder to prepare a steel slag aggregate. On the one hand, porous fly ash provides a channel for CO_2_ transport. On the other hand, lightweight fly ash reduces the weight of concrete. On this basis, the effects of different carbonation times and temperatures on carbonation products were studied using XRD and TG, and the mass of CO_2_ absorbed by the steel slag aggregate per unit mass was calculated in detail. The research results have positive significance for scientific carbon sequestration and the resource utilization of steel slag.

## 2. Raw Materials and Test

### 2.1. Raw Materials

**Figure 1:**
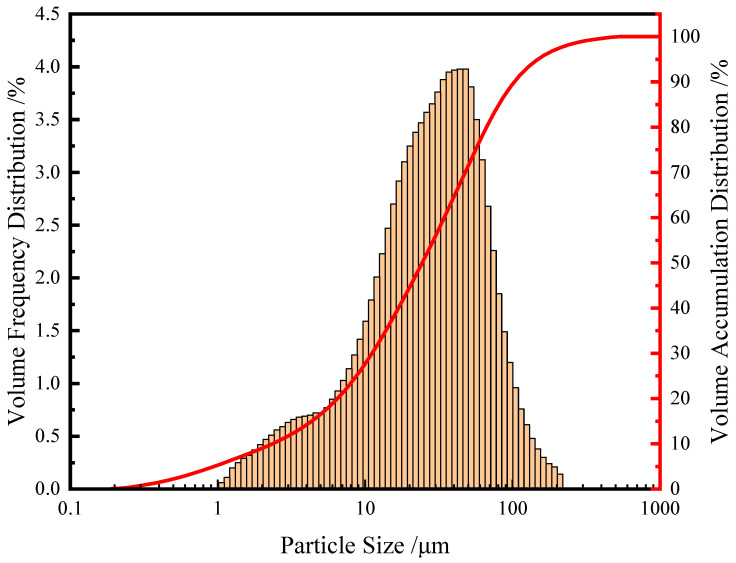
Particle size distribution of powdered steel slag.

**Figure 2:**
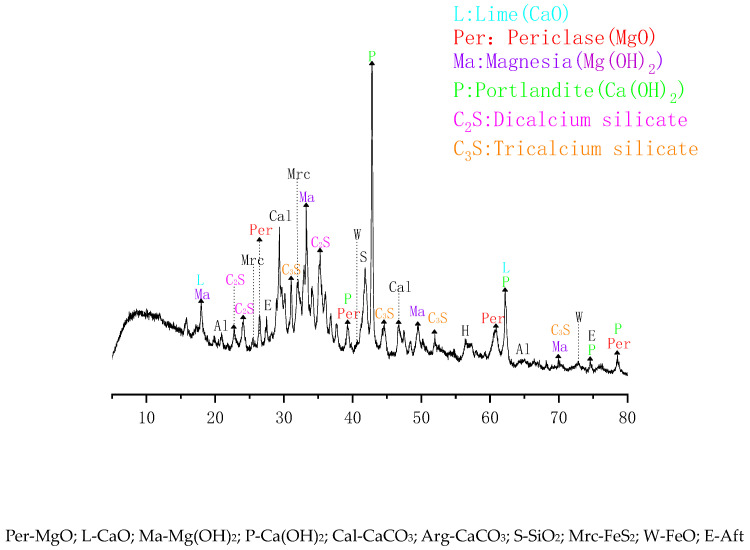
XRD pattern of steel slag.

The cement was P.O 42.5 Portland cement produced by Zhangjiagang Conch Cement Co., Ltd in Zhangjiagang, China. The fly ash was Class I fly ash produced by Shenhua Huashou Power Co., Ltd in Shanghai, China. The steel slag was provided by the Shagang Group in Suzhou, China. The steel slag powder was prepared by pulverizing the steel slag with a ball mill through a 0.08 mm sieve with a specific surface area of 260 m^2^/kg. Figure 1 shows the particle size distribution of steel slag after grinding. From Figure 1, it can be seen that the median particle size of powdered steel slag is 26.9 μm and the average particle size is 34.3 μm. The mineral composition of steel slag was tested using XRD. The results are shown in Figure 2. The chemical composition of raw materials is determined using the cement chemical analysis method, as shown in Table 1. Figure 2 shows that steel slag contains more CaO, followed by SiO_2_ and MgO, indicating that steel slag contains a large amount of basic oxide which can absorb and react with CO_2_.

### 2.2. Preparation

**Figure 3:**
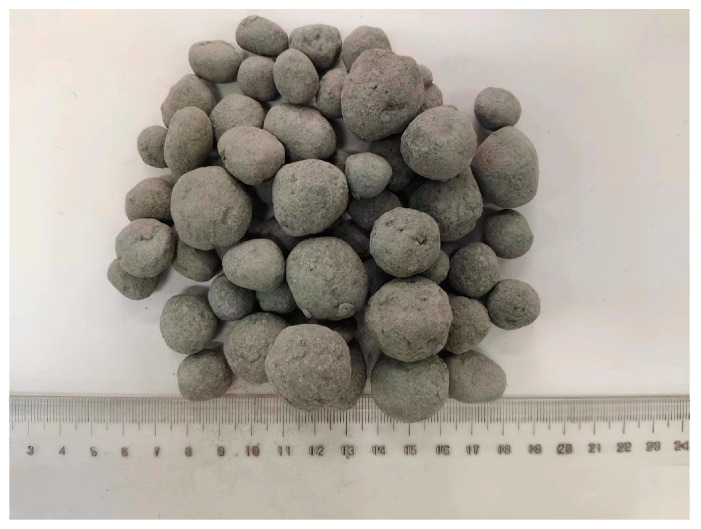
Steel slag aggregate.

The steel slag powder was the main component of this experiment, which was obtained by grinding steel slag through a ball mill and passing through a 0.08 mm sieve. The mixture of the aggregate was composed of steel slag powder, cement, and fly ash, with a ratio of 5:3:1. The steel slag was used as the main raw material, cement was used as cementitious material, and fly ash was added to reduce the quality of the aggregate and make an aggregate with a porous structure. The fly ash has two main functions. The first effect is to reduce the weight of the aggregate, which can effectively reduce the load on the foundation of the building. The second function is that the porous fly ash provides a channel for the transport of CO_2_, which can improve carbonation efficiency. The spherical steel slag aggregate was obtained by adding the abovementioned proportion into the pelletizer to mix evenly and spray-pelletizing. The particle size of the steel slag aggregate was 9–31.5 mm. The aggregate was placed in the pre-curing condition for 7 days (relative humidity 55 ± 5%, 20 ± 2 °C) and the uncarbonated steel slag was obtained (Figure 3).

**Figure 4:**
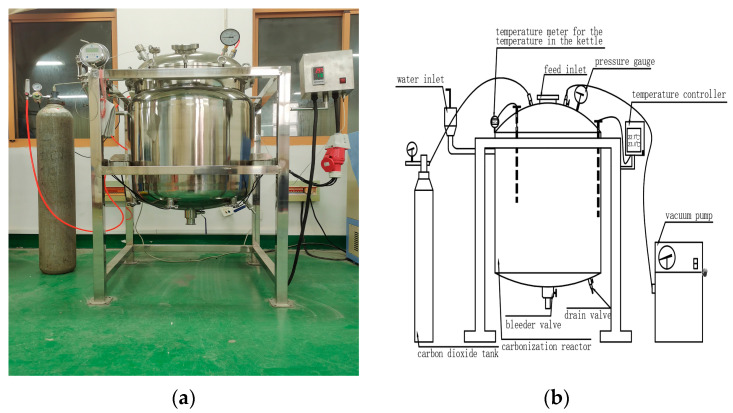
Schematic diagram of carbonation test: (**a**) Photo of test; (**b**) Setup for split tensile strength test.

The pre-cured steel slag aggregate was placed in a custom-made carbonation box with a carbon dioxide concentration of 99.9% and an ambient pressure of 0.2 MPa for carbonation curing. The schematic diagram of the carbonation test is shown in Figure 4. In order to determine the optimal carbonation time and carbonation effect at room and high temperatures, the carbonation temperature was set to 23 °C at normal temperature and 55 °C at high temperature for 3 h, 9 h, and 72 h, which were simplified as D1–D6, respectively. D0 was the blank control group without carbonation and granulation, as shown in Table 2.

### 2.3. Characterizations

#### 2.3.1. Volume Stability

**Figure 5:**
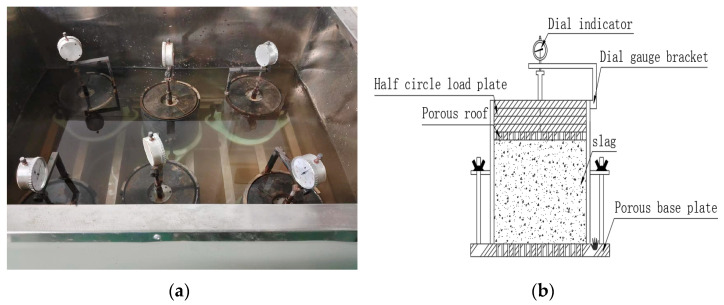
Measuring device for the swelling rate of immersion: (**a**) Photo of test; (**b**) Setup for split tensile strength test.

According to the specification of the steel slag stability test method (GB/T24175-2009) [32], the steel slag aggregate used in the test was determined using the method of water immersion expansion rate of steel slag as shown in Figure 5. The effect of different treatment methods on the expansibility of steel slag was evaluated using the expansion rate. In addition, the aggregates were autoclaved for 3 h at a temperature of 216 °C and pressure of 2 MPa. Its volume stability was determined by observing the damage of the steel slag aggregate particles.

#### 2.3.2. Crushing Value

The steel slag aggregate with a size range of 9.5–13.2 mm was utilized in the experiment, following the methodology for determining the crushing index value as stipulated in the test methods of highway engineering (JTG E42-2005) [33]. The mould containing the sample was placed on the press, and the pressure testing machine was started to reach a total load of 400 KN within 10 min, and the load was stabilized for 5 s. The finely crushed particles were removed by sieving with a diameter of 2.36 mm, and the mass left on the sieve was weighed to the nearest 1 g. The crushing index value Q was calculated according to Equation (1), accurate to 0.1%:

(1) $$Q=\frac{M_{1}}{M_{1}+M_{2}}\times 100\%$$

where *M*_1_ is the fine particle mass with a diameter less than 2.36 mm; *M*_2_ is the fine particle mass with a diameter greater than 2.36 mm.

#### 2.3.3. Product Analysis

The mineral composition of steel slag samples before and after carbonation was analyzed using X-ray diffraction (Rigaku SmartLab (3), Tokyo, Japan); the pattern was acquired between 5° and 80° with steps of 0.02°; the scanning speed was 10 °/min, and the mineral composition and degree of carbonation of the phase were determined using a thermogravimetric differential scanning calorimeter (Netzsch STA 449,Germany). For the mass absorption formula of carbon dioxide after carbonation stated in Formula (2), X is the amount of carbon dioxide absorbed by steel slag aggregates, while the CaCO_3_ formed is calculated from Formula (3) and Y is the amount of CaCO_3_ formed in steel slag aggregates [34]:

(2) $$X=\frac{G_{1}}{G_{2}}\times 100\%$$

(3) Y=G3−G4×10044×100%

where *G*_1_ is steel slag aggregate mass loss in 600–750 °C; *G*_2_ is steel slag aggregate mass at 900 °C; *G*_3_ is steel slag aggregate mass at 600 °C; *G*_4_ is steel slag aggregate mass at 750 °C.

## 3. Results and Discussion

### 3.1. Volume Stability

**Figure 6:**
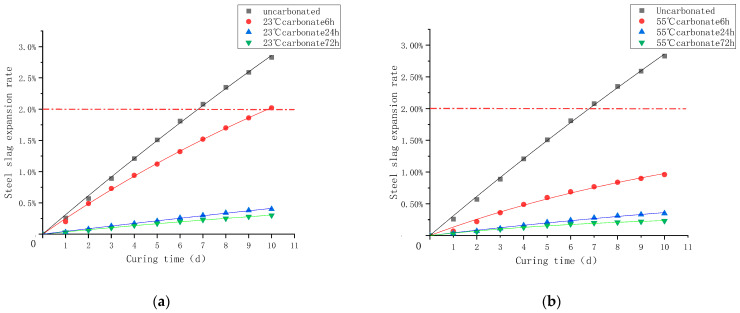
Daily expansion rate of steel slag aggregates. (**a**) carbonation temperature 23 °C; (**b**) carbonation temperature 55 °C.

The immersion expansion rate was measured for the steel slag aggregate, and the expansion rate of each group was plotted according to the results as shown in Figure 6; the expansion rate of steel slag aggregates with different carbonation times increases rapidly in the first three days but slowly thereafter whether at room temperature (23 °C) or a high temperature (55 °C). However, the expansion rate of uncarbonated D0 exceeds 2% at 7 d, which exceeds the prescribed limit. Except for group D1, which has an expansion rate of over 2% with a carbonation time of 6 h at 23 °C after 10 d, those of D2–D6 were 0.4%, 0.3%, 0.88%, 0.35%, and 0.23%, respectively, which was much less than that of the standard level.

The explanation for this phenomenon is that f-CaO content in the steel slag aggregate decreased obviously after the reaction at the high temperature and the long carbonation time, and the hydration product C-S-H gel was formed by C_2_S and C_3_S in the steel slag; this was equivalent to forming a protective shell on the surface of the steel slag, which hindered the further reaction between the steel slag and water and thus reducing the hydration reaction rate of the steel slag [35]. Furthermore, the expansion rate of the steel slag aggregate treated at 55 °C for 72 h is only 0.23%, which is 88.5% lower than the standard value and has the optimum expansion stability.

**Figure 7:**
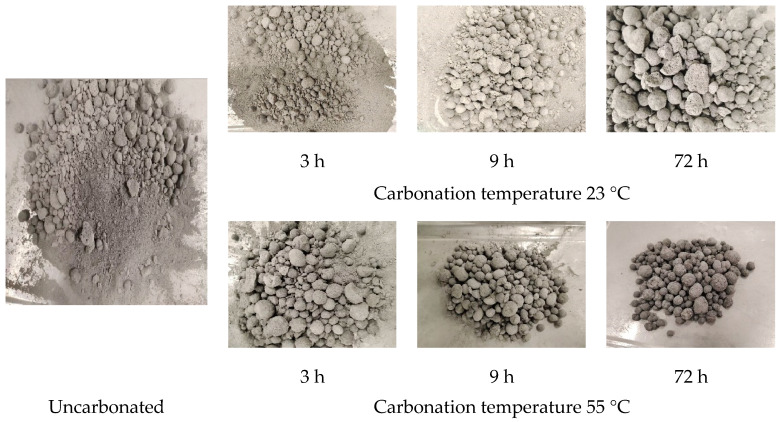
Photos of steel slag aggregates after the autoclaving test.

As shown in Figure 7, the steel slag aggregate that has not undergone carbonation treatment is completely pulverized after the autoclaving test. Mainly due to the large amount of f-CaO, f-MgO in the aggregate undergoes hydration and expansion under saturated steam conditions, ultimately leading to the crushing and pulverization of the aggregate. After the carbonation treatment, the shattered ratio of the steel slag aggregate improved. With the increase in carbonation time and carbonation temperature, the shattered ratio of the steel slag aggregate also decreased. This is because when the steel slag aggregate undergoes carbonation treatment, f-CaO reacts with CO_2_ and is consumed more without causing significant expansion. Under the carbonation curing condition of 55 °C for 72 h, the pulverization rate of steel slag aggregate was only 2.4% with almost no crushing (Table 3). This indicates that carbonation treatment can effectively improve the problem of poor volume stability caused by the expansion of steel slag aggregates. The prepared steel slag aggregate after carbonation treatment has a low expansion rate, good volume stability, can be better used in the building materials industry, especially in concrete and roadbed engineering, with good stability, and can be used to replace stones to a certain extent.

### 3.2. Crushing Value

**Figure 8:**
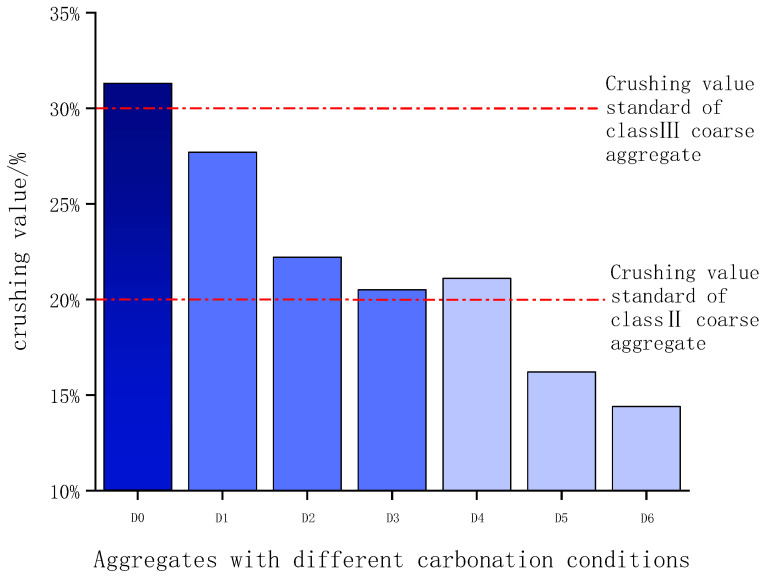
Crushing value of steel slag aggregates after carbonation treatment.

Figure 8 shows the crushing values of the steel slag aggregates with different carbonation times at 23 °C and 55 °C. At the same carbonation temperature, the crushing value of the aggregate decreases with the increase in carbonation time. The crushing values of the aggregates treated at a carbonation temperature of 55 °C are significantly lower than those at 23 °C. The crushing value of uncarbonated steel slag aggregate (D0) is 33.2%, which exceeds the limit for Grade III coarse aggregate in highway engineering (30%). When the carbonation temperature is 23 °C, only the crushing value of the steel slag aggregate carbonated for 72 h is less than 25%, satisfying the requirements for Grade III but not Grade II coarse aggregate yet. When the carbonation temperature increases to 55 °C, the crushing value of the steel slag aggregate cured for 72 h decreases to 23%, which is less than 25%, and reaches the standard requirement for Grade II coarse aggregate. Because the prepared steel slag aggregate had a porous structure and low strength, after carbonation curing, the generated calcium carbonate filled the pores, making the internal structure of the aggregate more dense, and the strength of the aggregate also increased.

### 3.3. Reaction Products

**Figure 9:**
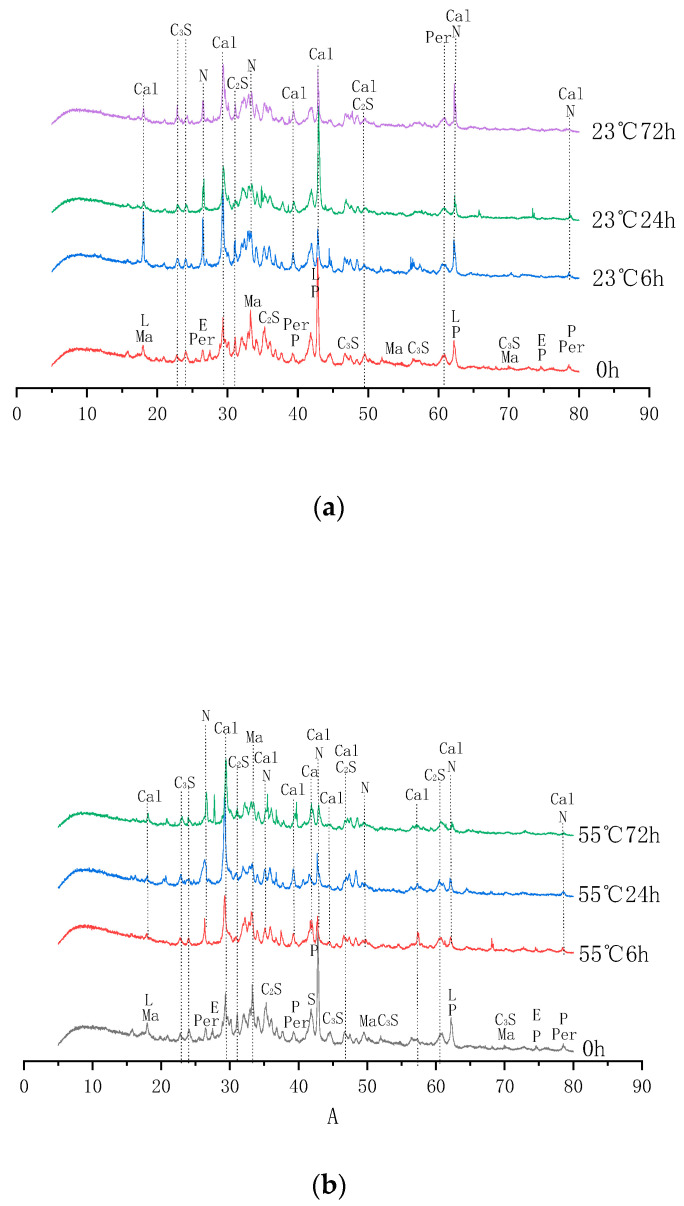
XRD analysis pattern of steel slag aggregates after carbonation curing. (**a**) carbonation temperature 23 °C; (**b**) carbonation temperature 55 °C.

As shown by the XRD pattern in Figure 9, the diffraction peak of C_2_S decreases and the diffraction peak of C_3_S and f-CaO disappears after carbonation as compared to the non-carbonated. In addition, the diffraction peak of Ca(OH)_2_ decreased with the increase in carbonation time, while the diffraction peak of CaCO_3_ was produced after carbonation and is obviously enhanced with the increase in carbonation temperature and carbonation time. The results confirmed that f-CaO, C_2_S, C_3_S, and Ca(OH)_2_ in the steel slag aggregate reacted with CO_2_ to form CaCO_3_, as the following equations show [36]:

(4) $$2\left(3CaO•SiO_{2}\right)+CO_{2}+3H_{2}O\to 3CaO•2SiO_{2}•3H_{2}O+{\mathrm{CaCO}}_{3}$$

(5) $$2\left(3CaO•SiO_{2}\right)+3CO_{2}+3H_{2}O\to 3CaO•2SiO_{2}•3H_{2}O+3{\mathrm{CaCO}}_{3}$$

(6) $$CaO+H_{2}O\to Ca{\left(OH\right)}_{2}$$

(7) $$Ca{\left(OH\right)}_{2}+CO_{2}\to H_{2}O+{\mathrm{CaCO}}_{3}$$

The results confirmed that as the carbonation temperature and carbonation time increased, more of the reaction product CaCO_3_ was generated and filled the pores in the aggregate, which enhanced the strength and decreased the crushing value of the aggregates.

**Figure 10:**
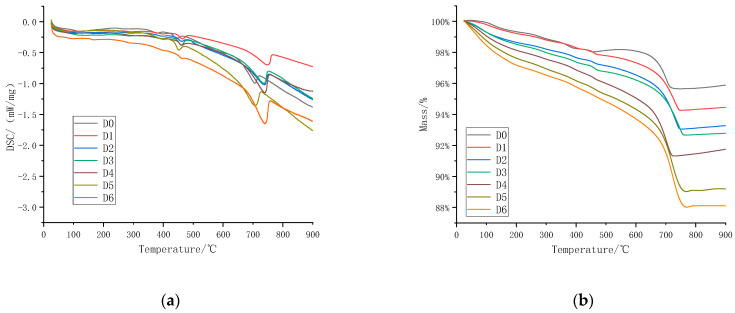
TG/DSC curve of steel slag aggregates after carbonation. (**a**) DSC curve; (**b**) TG curve.

In order to quantitatively analyze the CO_2_ absorption capacity of the steel slag aggregates with TG/DSC, the results are shown in Figure 10.

In Figure 10a, there are two distinct endothermic peaks, wherein the one within the temperature range of 400–450 °C can be ascribed to the decomposition of Ca(OH)_2_ and the endothermic peak in the temperature range of 600–750 °C resulted from the decomposition of CaCO_3_ [36,37]. The content of Ca(OH)_2_ and the endothermic peak of Ca(OH)_2_ decrease with the increase in carbonation temperature and carbonation time. In particular, the endothermic peak of the steel slag aggregate disappears almost completely after carbonation at 55 °C for 72 h, while the endothermic peak becomes more and more obvious in the temperature range of 600–750 °C, which indicates that more and more CaCO_3_ was produced during carbonation curing.

Compared to other studies where steel slag was directly carbonated for 24 h, the carbon dioxide absorption was only 7.09 wt.% [36]. In this study, the carbon dioxide absorption of steel slag after carbonation treatment after grinding is much higher than the conclusion in the literature. The CO_2_ absorption and CaCO_3_ production of steel slag aggregate under carbonation curing were determined according to the TG/DSC curves listed in Table 4. It can be seen that the steel slag aggregate absorbs 6.76–9.04 wt.% CO_2_ after carbonation. As the carbonation time increases, the amount of CO_2_ absorption also increases. The steel slag aggregate absorbs an additional 7.4–8.74 wt.% CO_2_ when the carbonation time increases from 0 to 72 h. When the carbonation time increases from 6 h to 72 h, the aggregate absorbs an additional 2.28 wt.% and 1.34 wt.% CO_2_ at room temperature and high temperature, respectively. It can be seen that the carbonation efficiency of steel slag aggregate in 0–24 h is 1.89 times that of 24–72 h. This is due to the reaction of CO_2_ with f-CaO, f-MgO, and so on during carbonation. Decreasing calcium content can take part in the reaction. In addition, with the formation of the carbonation product CaCO_3_, the pores in the aggregate are filled up, remarkably boosting the enhancement of structural densification, which is unfavorable to the penetration of CO_2_ gas and hinders the carbonation reaction; the efficiency of early carbonation is higher than that of late carbonation. From the tests above, not only will the carbonation temperature and time affect the carbonation efficiency of the steel slag aggregate but the composition and structure of the steel slag aggregate will also play a key role in carbonation. In addition, the steel slag aggregate shows different carbonation efficiencies at different carbonation periods.

## 4. Conclusions

This study not only reduced the weight of steel slag aggregate but also improved its carbonation efficiency by mixing powdered steel slag with fly ash. The reaction products of steel slag were analyzed using XRD and TG, and based on this, the carbon sequestration efficiency under different curing methods was calculated. The research results provide a solution for the resource utilization of steel slag and a theoretical calculation basis for scientifically reducing “carbon emissions”. Based on the experimental results, the following conclusions can be drawn:

(1) Untreated steel slag contained a large amount of active CaO, with a pulverization rate of 55.8% and a crushing value of 33.2%. When used directly, it will cause harmful expansion and damage the integrity of the aggregate. Therefore, untreated steel slag cannot be used to prepare steel slag concrete.
(2) Under high temperatures and pressure, the steel slag could quickly react with CO_2_ to generate CaCO_3_, which filled the voids in the aggregate and improved its performance. This indicates that fly ash could not only reduce the weight of the aggregate but also provide channels for CO_2_ and improve carbonation efficiency. This proves that the process of producing artificial aggregates by mixing powdered steel slag, fly ash, and cement in this study was reasonable. After comparison, the best carbonation method was to cure at 55 °C for 72 h. The pulverization rate of the carbonated steel slag aggregate was 2.4%, the expansion rate was 0.23%, and the crushing value was 23%, meeting the requirements of Class II aggregate.
(3) The carbon sequestration efficiency was closely related to temperature and time, and increasing the curing temperature and time could effectively improve the carbon sequestration efficiency of steel slag. When the curing temperature was 23–55 °C and the curing time was 6–72 h, the carbon sequestration efficiency was increased from 6.76% to 11.27% (in mass fraction).

## Figures and Tables

**Table 1:** Chemical compositions of raw materials.

Raw Material	Mass Fraction									
	CaO	MgO	SiO_2_	Al_2_O_3_	Fe_2_O_3_	K_2_O	Na_2_O	SO_3_	LOSS	Total
Cement	63.95	2.40	19.40	4.76	2.94	0.66	0.12	2.59	2.83	99.65
Steel Slag	41.78	10.72	9.75	2.60	29.25	0.04	0.08	0.07	1.55	95.84
Fly Ash	3.96	1.06	48.82	27.84	6.10	1.14	0.40	0.94	6.13	96.39

**Table 2:** Carbonation test grouping.

Test Group	D0	D1	D2	D3	D4	D5	D6
Carbonation temperature (°C)	-	23	23	23	55	55	55
Carbonation time (h)	-	3	9	72	3	9	72

**Table 3:** The pulverization rate of steel slag aggregate.

Test Group	D0	D1	D2	D3	D4	D5	D6
pulverization rate (%)	55.8	17.7	9.8	4.4	11.2	3.5	2.4

**Table 4:** CO_2_ absorption and CaCO_3_ content in steel slag aggregate after carbonation curing.

Test Group	600–750 °C Mass Loss/mg	CO_2_ Absorption/wt.%	CaCO_3_ Content/wt.%
D0	0.46	2.53	1.05
D1	3.78	6.76	8.58
D2	5.52	7.57	12.55
D3	6.95	9.04	15.80
D4	6.76	9.93	15.36
D5	7.49	10.13	17.01
D6	9.13	11.27	20.74

## Data Availability

All data generated or analyzed in this research were included in this published article. Additionally, readers can access all data used to support conclusions of the current study from the corresponding author upon request.
